# Low-frequency attenuation filter as a reversible cause of inappropriate shocks due to myopotential oversensing

**DOI:** 10.1016/j.hrcr.2021.05.016

**Published:** 2021-05-27

**Authors:** Peysh A. Patel, Ramesh Nadarajah, Sam Straw, Klaus K. Witte

**Affiliations:** Department of Cardiology, Leeds General Infirmary, Leeds, United Kingdom

**Keywords:** Device troubleshooting, Implantable cardioverter-defibrillator, Inappropriate shocks, Low-frequency attenuation filter, Myopotential oversensing

## Introduction

Inappropriate device shocks from implantable devices can be owing to supraventricular dysrhythmias, intracardiac sensing (ie, P, R, or T waves), or extracardiac detection secondary to noise from lead fracture, myopotentials, or electromagnetic interference.^1^ When device malfunction occurs, a rigorous exploration of programming algorithms is warranted as part of the diagnostic strategy. We present the timeline and progress of a middle-aged man who presented with recurrent inappropriate shocks owing to myopotential oversensing. This was eradicated in its entirety by simply turning “off” a specific feature that attenuates low-frequency signals, and would have prevented an unnecessary lead revision for perceived failure.

## Case report

A 61-year-old man was admitted to his district general hospital with dizziness. His past medical history was relevant for ST-elevation myocardial infarction with prior left anterior descending percutaneous intervention, peripheral vascular disease, and unprovoked deep vein thrombosis and pulmonary embolus, for which he was maintained on lifelong rivaroxaban. On initial assessment, he was found to be in monomorphic ventricular tachycardia with cycle length of 325 ms requiring synchronized electrical cardioversion. There were 3 further similar events during admission, despite high-dose bisoprolol and intravenous amiodarone. Transfer to the local tertiary center was arranged.

A 12-lead electrocardiogram confirmed presence of anterior Q waves and broad left bundle branch block (QRS 152 ms), and echocardiography revealed severe reduction of left ventricular systolic function with an ejection fraction of <35%. Blood profiling showed normal inflammatory markers, electrolytes, and thyroid function, with raised troponin. COVID swab returned negative. Coronary angiography excluded flow-limiting disease. Radiofrequency ablation of an anteroapical scar was performed and in view of pre-existing indication being aligned with existing European Society of Cardiology (ESC) guidelines,^2^ he also received a cardiac resynchronization therapy with defibillator (CRT-D) device after appropriate counseling. This was performed via a traditional subclavian approach, with positioning of a single-coil right ventricular (RV) lead at the apex (Abbott Durata 7120Q, Sylmar, CA), right atrial lead at the appendage (Abbott Tendril STS 2088TC, Sylmar, CA), and left ventricular lead in a high lateral target vein (Abbott Quartet 1458QL, Sylmar, CA). The RV lead was set in a true bipolar sensing configuration, with R-wave sensing of 11.5 mV at time of implant and an adequate current of injury. The leads were attached to an Abbott Quadra Assura generator (Sylmar, CA). Subsequent chest radiograph and repeat device checks were satisfactory, resulting in prompt discharge.

Readmission occurred after the patient experienced 2 shocks from his device while asleep. He denied preceding chest pain, palpitations, dizziness, or syncope. Interrogation confirmed a drop in R-wave sensing close to the lowest nominated programmed value of 0.5 mV; and 60 “VF [ventricular fibrillation] episodes,” of which 44 were interpreted as noise owing to RV oversensing. This correlated with a drop in biventricular pacing percentage to 72%. There were 2 further device shocks during admission; repeat chest radiography did not suggest lead macro-displacement. Nonetheless, in the absence of clear reversible etiology, the lead was repositioned to a distant apical site. All parameters were satisfactory aside from a mildly high stimulation threshold (1.7 V at 0.5 ms), which was accepted.

**Figure 1:**
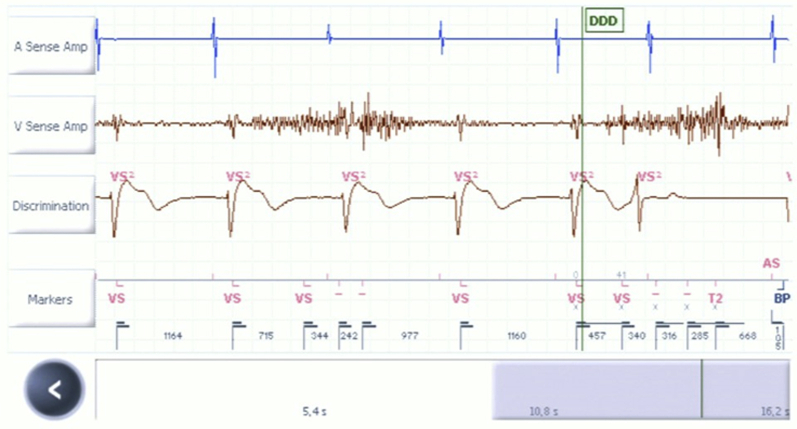
Real-time electrogram with nominal sensitivity settings and low-frequency attenuation filter programmed “on.” Right ventricle sense amplifier displays high-frequency, low-amplitude signals with a respirophasic pattern suggestive of myopotentials. These are oversensed and marked as “VS.” Sweep speed 25 mm/s.

**Figure 2:**
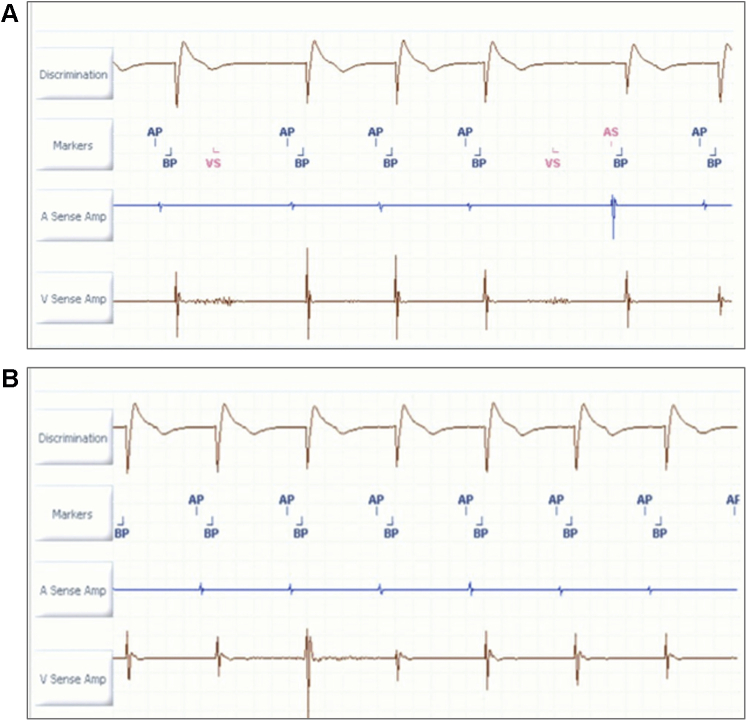
Panel comparing electrograms (EGMs) with and without low-frequency attenuation (LFA) filter. **A:** Real-time EGM depicts noise on ventricular channel during isometric testing suggestive of diaphragmatic myopotential oversensing. **B:** Deactivation of LFA filter results in elimination of lead noise with appropriate sensing. Sweep speed 25 mm/s.

A subsequent patient recall was necessitated at 4 days after remote download alerted to recurrence of RV oversensing secondary to noise (Figure 1). At this point, the manufacturer’s diagnostics team (Abbott) was contacted. As other lead parameters were adequate, a search for alternative causes was prompted prior to second reposition. RV lead screening under fluoroscopy in multiple imaging planes excluded gross abnormalities in lead integrity or positioning. However, lead noise was reproducible during deep inspiration and coughing, with appearances consistent with diaphragmatic myopotential oversensing. Based on advice from the diagnostics team, the low-frequency attenuation (LFA) filter was turned from “on” (ventricular sensitivity 0.5 mV, threshold start 50%, decay delay 0 ms) to “off” (ventricular sensitivity 0.3 mV, threshold start 62.5%, decay delay auto). This eliminated lead noise in its entirety, with unremarkable isometric testing (Figure 2). No other programming changes were required and the patient was discharged. He has been clinically stable since, with no further reported therapies from his device after 6 months of follow-up.

## Discussion

**Figure 3:**
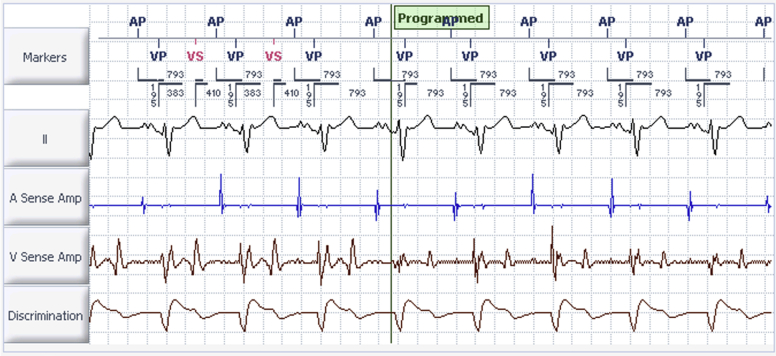
Comparison of electrograms based upon low-frequency attenuation (LFA) filter settings. Initial deactivation results in larger-amplitude T-wave signals, which are occasionally oversensed (depicted by “VS”). Subsequent reprogramming and activation of LFA filter results in marked reduction in amplitude of T-wave signal and elimination of T-wave oversensing. Sweep speed 25 mm/s.

LFA filters are a strategic programming feature of contemporary Abbott devices that have defibrillator capability.^3^ The primary objective of this filter is to mitigate sequelae arising from T-wave oversensing (ie, inappropriate device shocks). When it is programmed “on,” the filter attenuates the amplitude of such low-frequency signals (see Figure 3). However, as with any bandpass filter, this can amplify other event frequencies, including high-frequency, low-amplitude signals arising from sources such as myopotentials and electromagnetic interference.^4^ If the amplitude of these signals meets a specified threshold, it is large enough to be sensed by the device. This is illustrated in our case, where diaphragmatic myopotential oversensing occurred during isometric testing and could be terminated simply by turning the filter “off.” A similar phenomenon has been demonstrated previously in a case series of 2 patients with CRT-D implants, in which respiratory maneuvers resulted in myopotential oversensing that was exacerbated by the LFA filter and resulted in failure of pacing output.^5^

The LFA filter is nominally programmed “on.” This may decrease the ability to detect low-amplitude signals such as ventricular fibrillation, as the nominal maximum R-wave sensitivity is 0.5 mV, compared to 0.3 mV when programmed “off.” However, caution is equally warranted in deactivation of the feature, as it can result in decreased R-wave amplitude and more prominent T-wave amplitude with the inherent risk of oversensing. A comparable feature present in Medtronic devices is the T-wave discrimination algorithm. This uses frequency content to determine whether an intrinsic ventricular sensed event (ie, Vs on the marker channel) is due to the R wave or T wave. If it is the latter, it is labeled as “TW” and therapy is disabled. Hence, this manufacturer-specific feature does not truly eliminate T-wave oversensing but instead results in detection and labeling to withhold subsequent shocks.

In this case, detection of lead noise appeared to be concurrent with a drop in R-wave sensing, albeit without evidence of clear macro-displacement. Autosensing measurements depict the median value from 5 discrete measurements, however, so incorporation of low-amplitude signals from noise would skew derived values rather than reflecting a true reduction in R wave *per se*. Importantly, there were multiple separate episodes of lead noise detected by the device, but not all translated into inappropriate therapy. Additional programming features are available to directly address this phenomenon. For instance, all manufacturers have a noise reversion algorithm with labeling of signals if they exceed physiological rates (typically 400–600 beats/min).^6^ Once this counter is met, the device reverts to an asynchronous mode where sensing is entirely eliminated. A separate feature specific to Abbott systems is the SecureSense algorithm.^7^ This works on the premise that true noise secondary to a lead fracture would be detected by the near-field channel (eg, RV tip-ring) but not far-field channel (eg, RV coil-can). Hence, if there is rate inequality, the device will label this as RV oversensing and inhibit therapies. After 5 such episodes, an automatic alert will be sent, as in this case, prompting the need for formal assessment and investigation.

## Conclusion

This case seeks to highlight the clinical relevance of the LFA filter as a reversible cause of inappropriate shocks owing to diaphragmatic myopotential oversensing. Further exploration is warranted to assess prevalence and clinical burden, as unnecessary lead revision may be avoided by simple reprogramming and filter deactivation.

**Key Teaching Points**

• This case highlights the need to be familiar with programming features specific to different device manufacturers.
• The low-frequency attenuation filter in Abbott devices is nominally programmed “on” and alters sensing bandpass to prevent T-wave oversensing.
• However, it can amplify high-frequency signals such as diaphragmatic myopotentials.
• Awareness of this may prevent otherwise unnecessary lead revisions for perceived failure.
